# Supplementary material for: Nrl knockdown by AAV-delivered CRISPR/Cas9 prevents retinal degeneration in mice
Source: Nat Commun. 2017 Mar 14;8:14716. doi: 10.1038/ncomms14716 (PMC5355895; doi:10.1038/ncomms14716)
Supplement: Supplementary Information — Supplementary figures, supplementary tables and supplementary references. [file ncomms14716-s1.pdf]

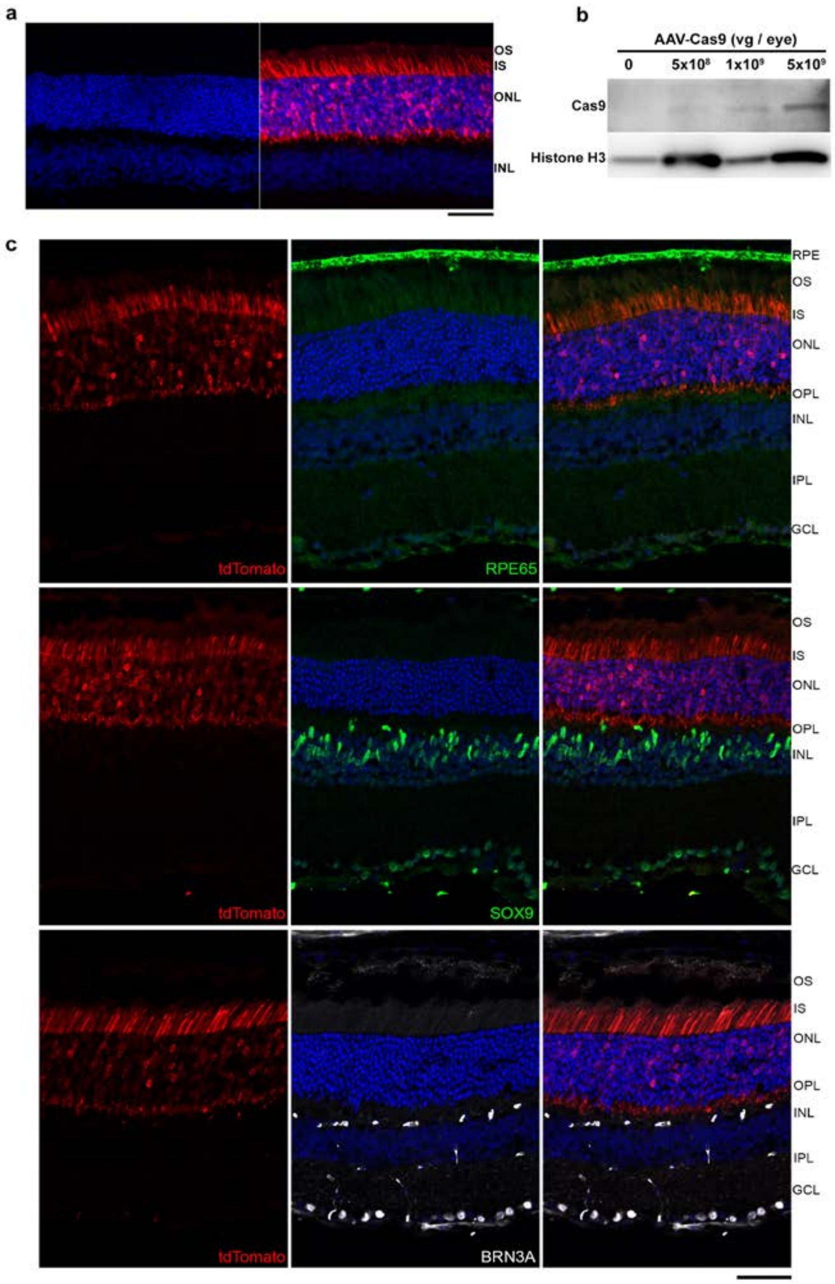

Supplementary Figure 1. AAV-mediated CRISPR/Cas9 transduction in mouse retina. Vector administration was performed when mice were 2 months old. **(a)** Expression of tdTomato (red) in photoreceptors in a C57bl/6j mouse at 6 weeks after subretinal injection of 2.5×10^{9} vg AAV-sgRNA. Dapi is shown in blue. **(b)** Immunoblot analysis of SpCas9 protein in the retinas isolated from two mice (including both genders) receiving subretinal injection of AAV-Cas9 with different doses. Vehicle injection was served as negative controls. Histone H3 was served as loading controls. **(c)** Immunostaining for RPE65, SOX9 or BRN3A for a male C57bl/6j mouse at 6.5 months after receiving co-administration of AAV-Cas9 and AAV-sgRNA-EGFP. Expression of tdTomato was not observed in RPE or other retinal layers. RPE, retinal pigment epithelium; OS, outer segments; IS, inner segments; ONL, outer nuclear layer; OPL, outer plexiform layer; INL, inner nuclear layer; IPL, inner plexiform layer; GCL, ganglion cell layer. Scale bars: 50 μm.

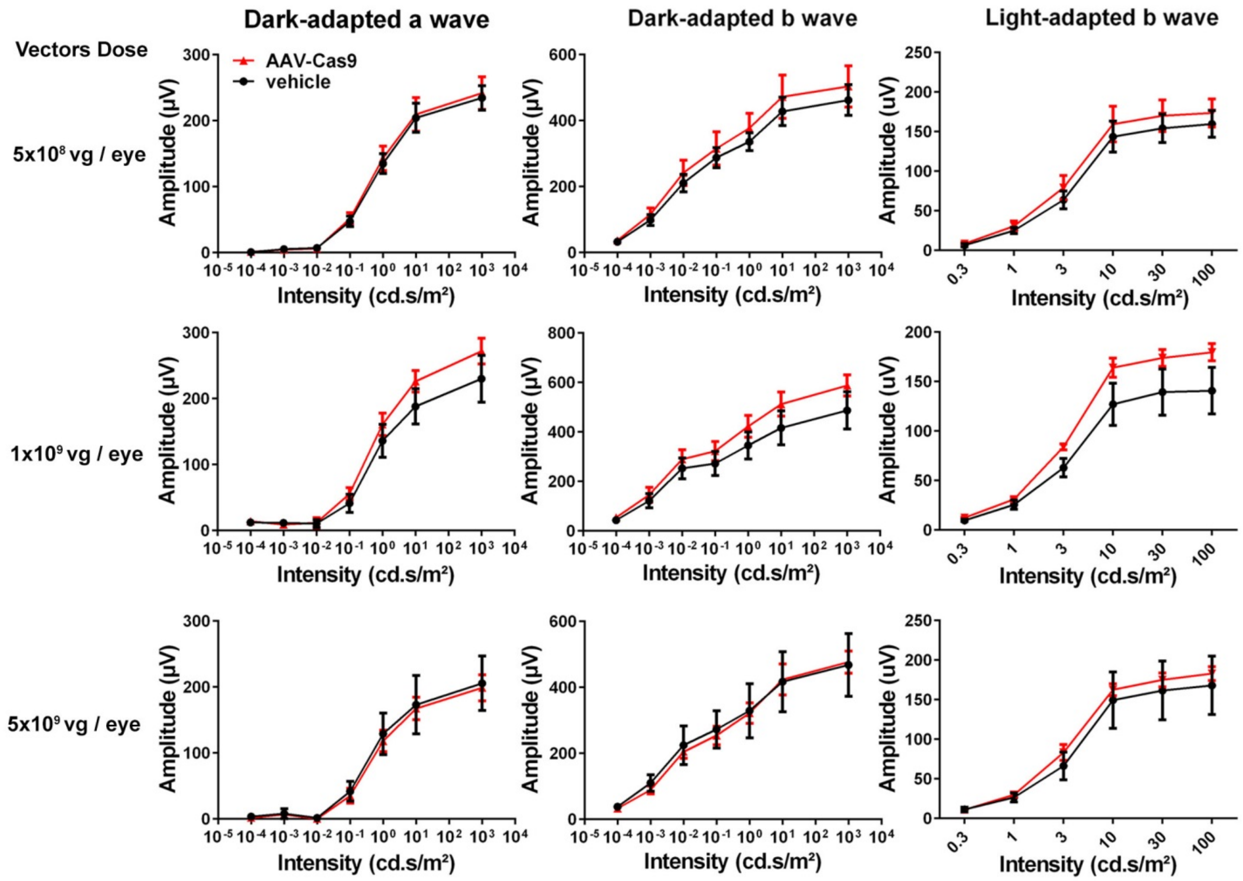

Supplementary Figure 2. ERG analyses of C57bl/6j mice treated with AAV-Cas9 vector. Each mouse received subretinal administration of the vector in one eye and vehicle in the fellow eye at 1 month of age. ERG was conducted at 4 months post treatment. The significance between the vector- and vehicle-treated eyes was calculated using two-tailed paired t-test. Five male mice were used for each dose. No significant difference (P>0.05) was observed in either dark-adapted or light-adapted ERG between the vector- and vehicle-treated eyes.

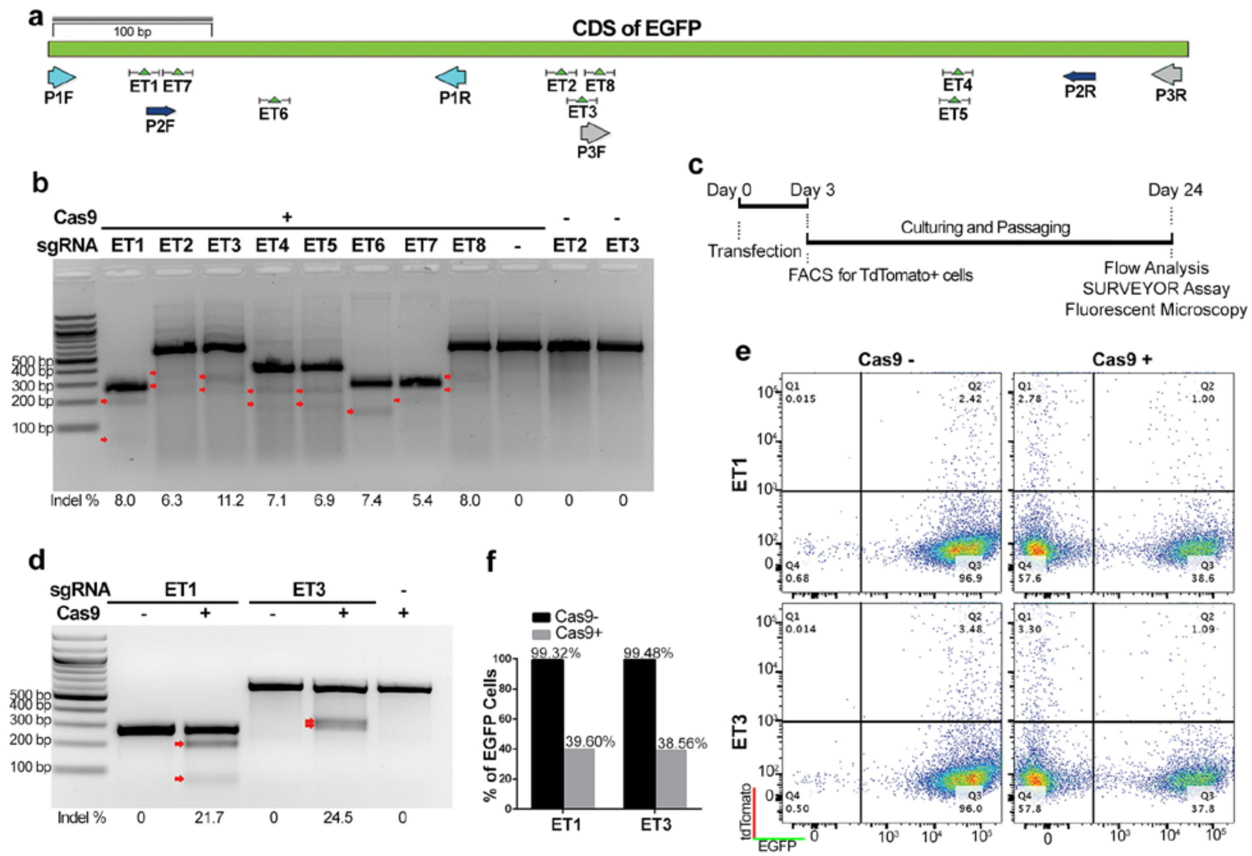

Supplementary Figure 3. CRISPR/Cas9 mediated EGFP gene disruption in HEK-293 cells. (a) Schematic of the locations of eight candidate sgRNA targets and three pairs of PCR primers in EGFP coding sequence (CDS). **(b)** SURVEYOR nuclease assay following co-transfection of the construct carrying CMV promoter-driven Cas9 with each of the eight sgRNA constructs into EGFP-integrated HEK-293 cells. DNA fragments digested by SURVEYOR nuclease are indicated by red arrows. Indel rate of each sample is shown below the gel image. **(c)** Timeline of the comparison of sgRNAs containing ET1 and ET3 protospacer sequences. **(d)** SURVEYOR nuclease assay after extended culturing of tdTomato-enriched HEK-293/EGFP cells for comparison of sgRNAs containing ET1 and ET3. **(e)** FACS plots. **(f)** Knockdown efficiency of sgRNAs containing ET1 and ET3.

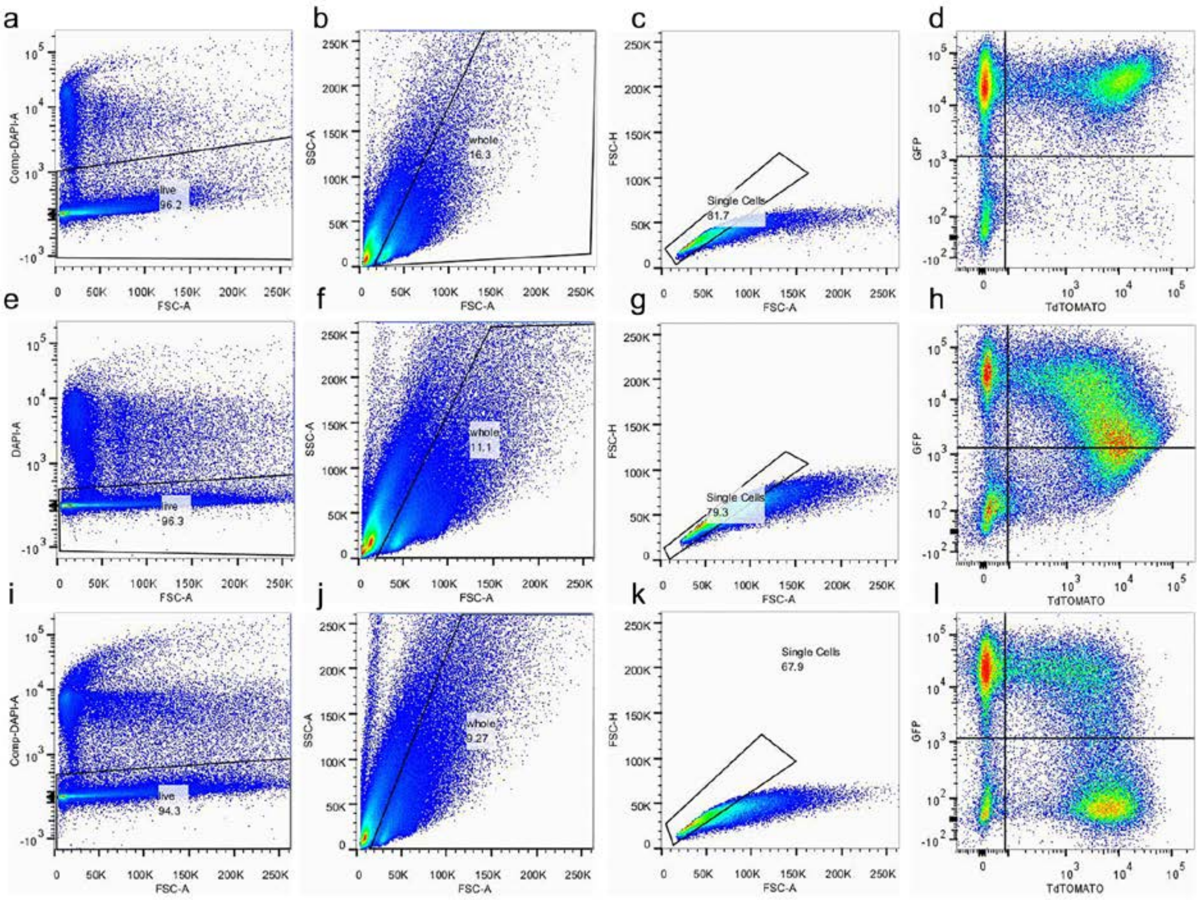

Supplementary Figure 4. Flow cytometry gating strategy for FACS of photoreceptors. Representative data from a control eye (**a–d**), a CRISPR-EGFP treated eye at 6 weeks post injection (**e-h**) and a CRISPR-EGFP treated eye at 10-week post injection (**i-l**) are shown. Whole live cells from dissociated retina cell suspension were gated firstly on a forward scatter (FSC-A)/ DAPI dot plot (**a, e, i**) and secondly on a forward scatter (FSC-A)/ side scatter (SSC-A) dot plot (**b, f, j**). Single cells were further gated on a FSC-A/FSC-H dot plot (**c, g, k**). Finally, the single cells were analyzed for EGFP/tdTomato expression (**d, h, l,** same as shown in Fig. 1e).

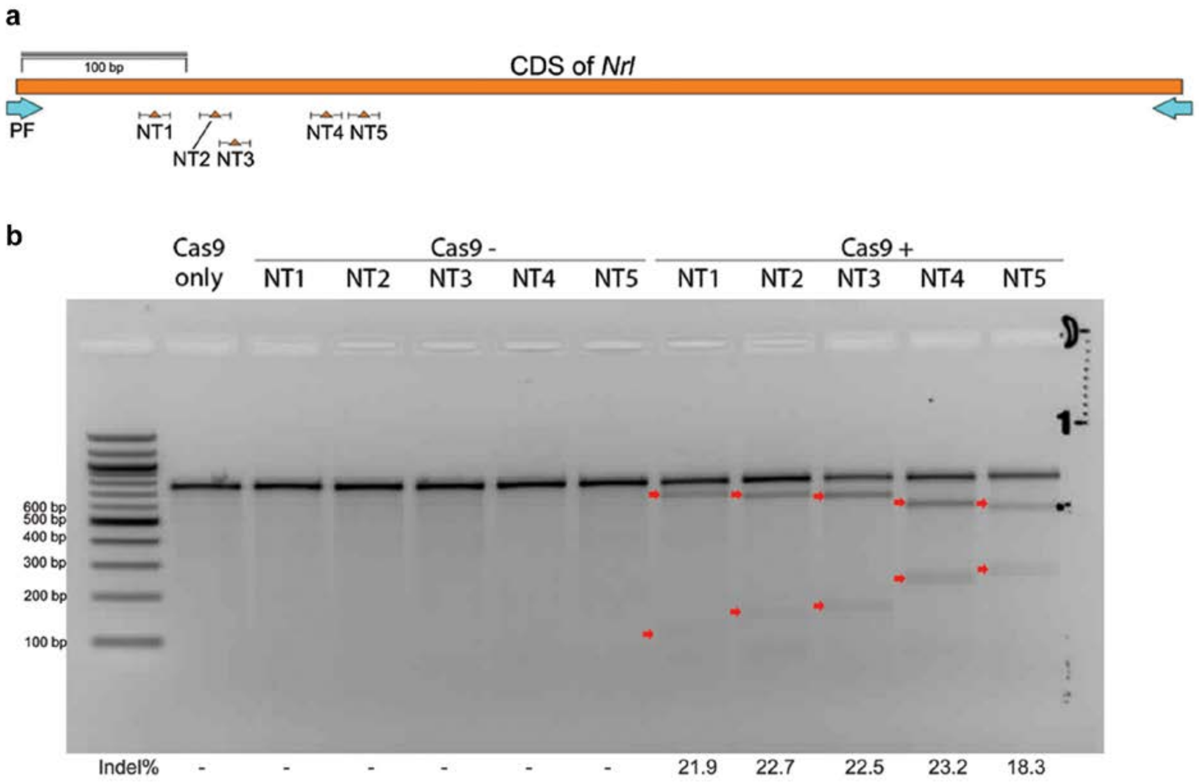

Supplementary Figure 5. *In vitro* screening of sgRNA candidates against *Nrl* gene. (**a**) Schematic of the locations of five candidate sgRNA targets and a pair of PCR primers in *Nrl* coding sequence (CDS). **(b)** SURVEYOR nuclease assay following co-transfection of each sgRNA construct with CMV promoter-driven Cas9 and *Nrl* cDNA constructs into HEK-293 cells. DNA fragments digested by SURVEYOR nuclease are indicated by red arrows. Indel rate of each sample is shown below the gel image.

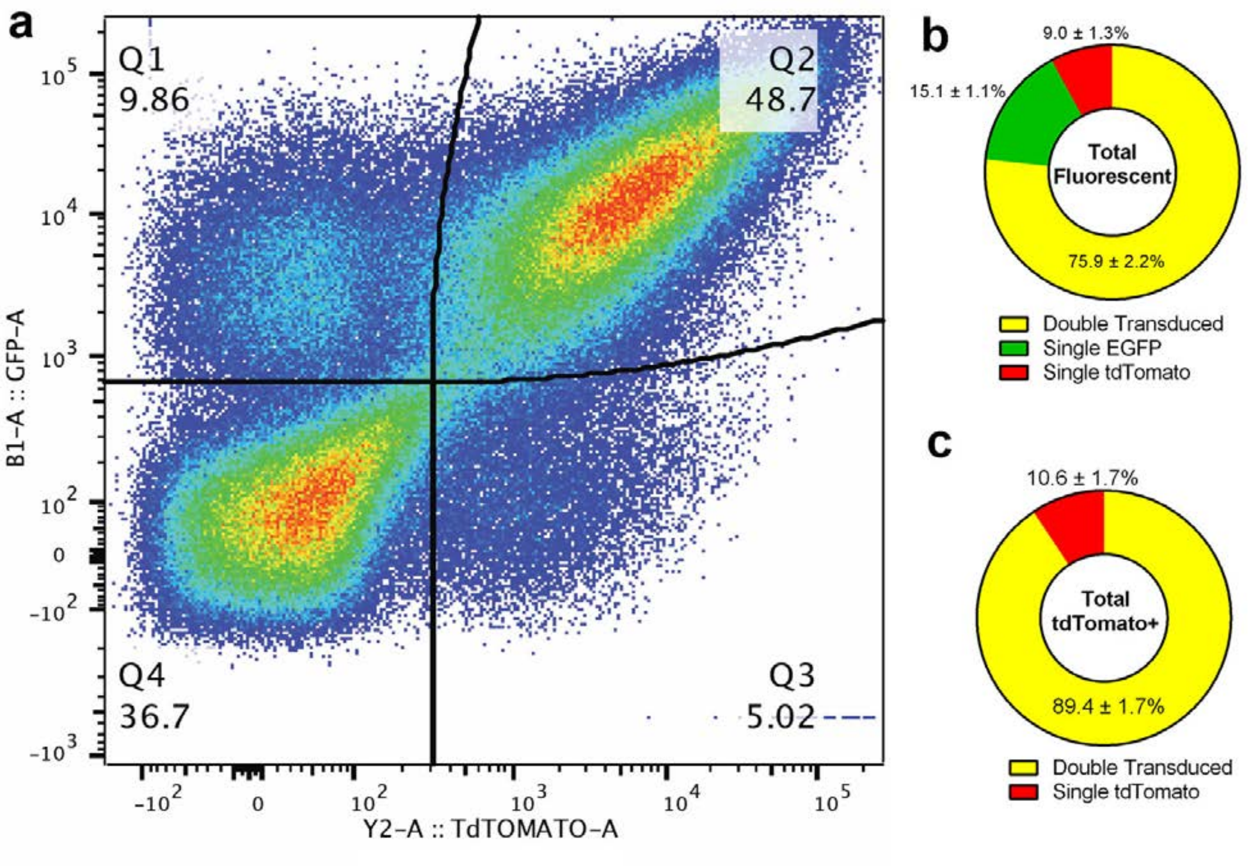

Supplementary Figure 6. Co-transduction of retinal cells by two AAV reporter vectors. Four C57bl/6j mice including both genders were subretinally co-injected with 5×10^9 vg AAV-RK-EGFP and 2.5×10^9 vg AAV-sgRNA vectors at P14. The AAV-sgRNA vector contains an RK promoter-driven tdTomato expression cassette. Flow cytometry analyses were conducted at 1 month post injection. (**a**) Representative FACS plots**.** (**b** and **c**) Statistical analysis showing the percentage of co-transduced cells in (**b**) total transduced cells and (**c**) AAV-sgRNA transduced cells (n = 4). Data (**b** and **c**) are represented as mean±s.d.

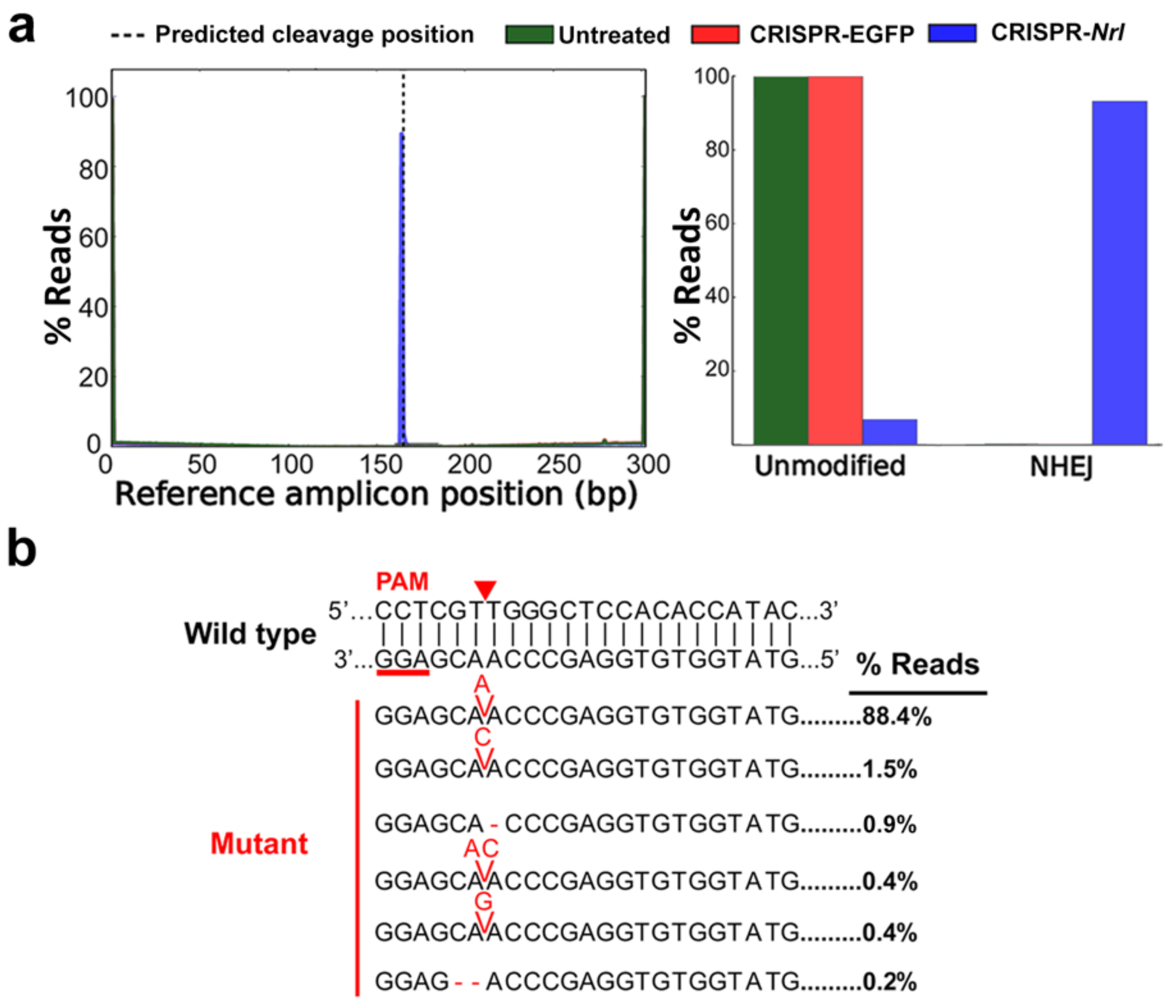

Supplementary Figure 7. Long-term on- and off-target effect following CRISPR-*Nrl* treatment. C57bl/6j mice (one male and one female) received subretinal vector administration at P14. Targeted DNA deep sequencing of mouse photoreceptors was performed at 10 months after treatment. **(a)** Rate of sequence change at the target site of *Nrl* locus in flow-sorted tdTomato-expressing cells. Schematic graphs indicate positions of indels in the amplicon (**left**) and total NHEJ frequencies (**right**) of untreated (green), CRISPR-EGFP treated (red) and CRISPR-*Nrl* treated eyes (blue). **(b)** Representative mutation patterns and corresponding ratios in total reads detected by deep sequencing of *Nrl* locus following CRISPR-*Nrl* treatment. Top, wild-type sequence. Red dashes, deleted bases; Red bases, insertions; Red triangle, CRISPR/Cas9 cutting site.

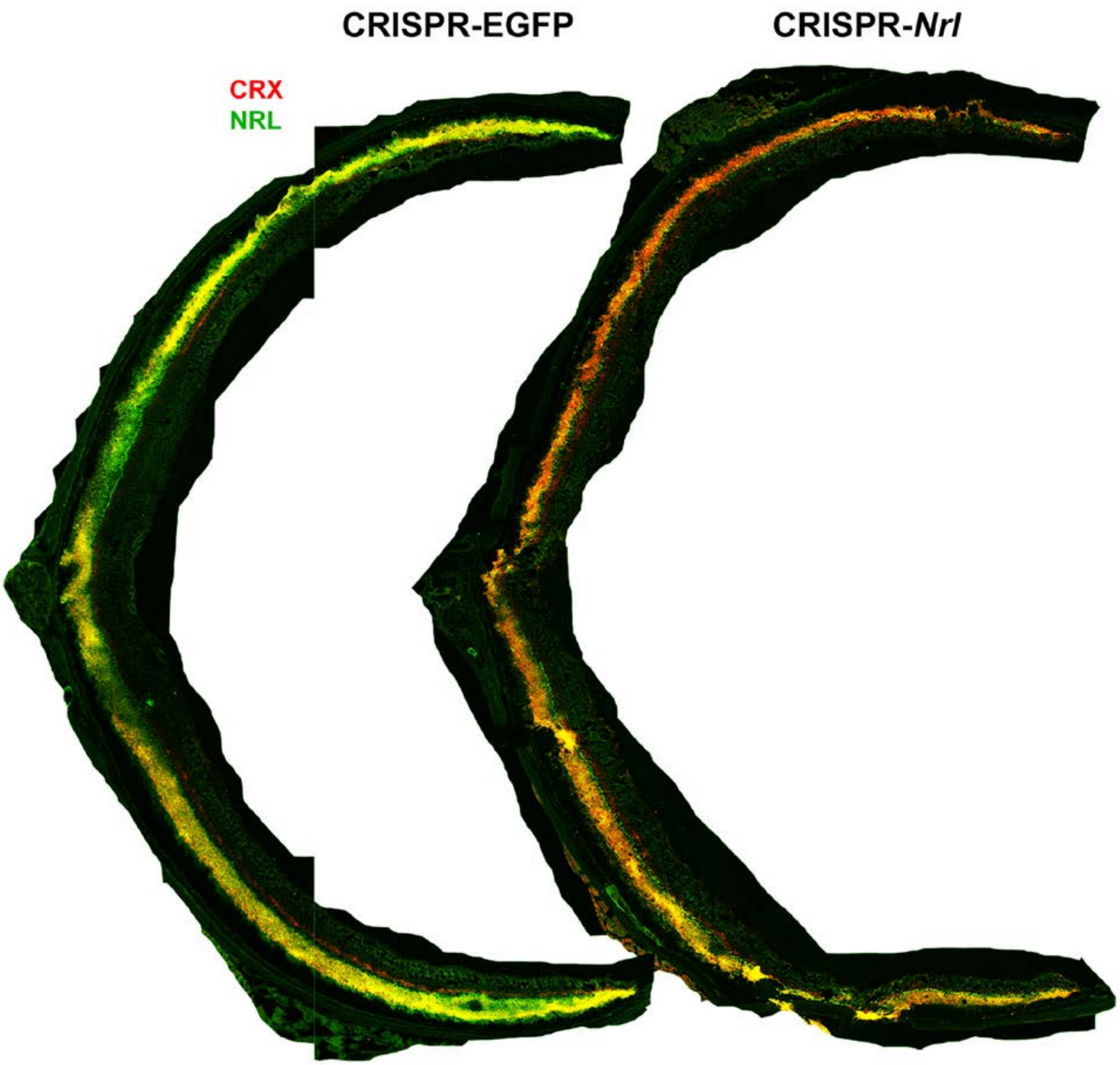

Supplementary Figure 8. AAV-CRISPR/Cas9 mediated *Nrl* knockdown in mouse retina. C57/Bl6 mice received subretinal administration of CRISPR-*Nrl* vectors in right eyes and the control CRISPR-EGFP vectors in left eyes at P14. Immunostaining for CRX and NRL in retinal sections of a P90 mouse is shown. CRX (red) is expressed in photoreceptors and was not affected by vector treatment in both eyes. In contrast, NRL expression (green) was absent in most of the retinal area following CRISPR-*Nrl* treatment.

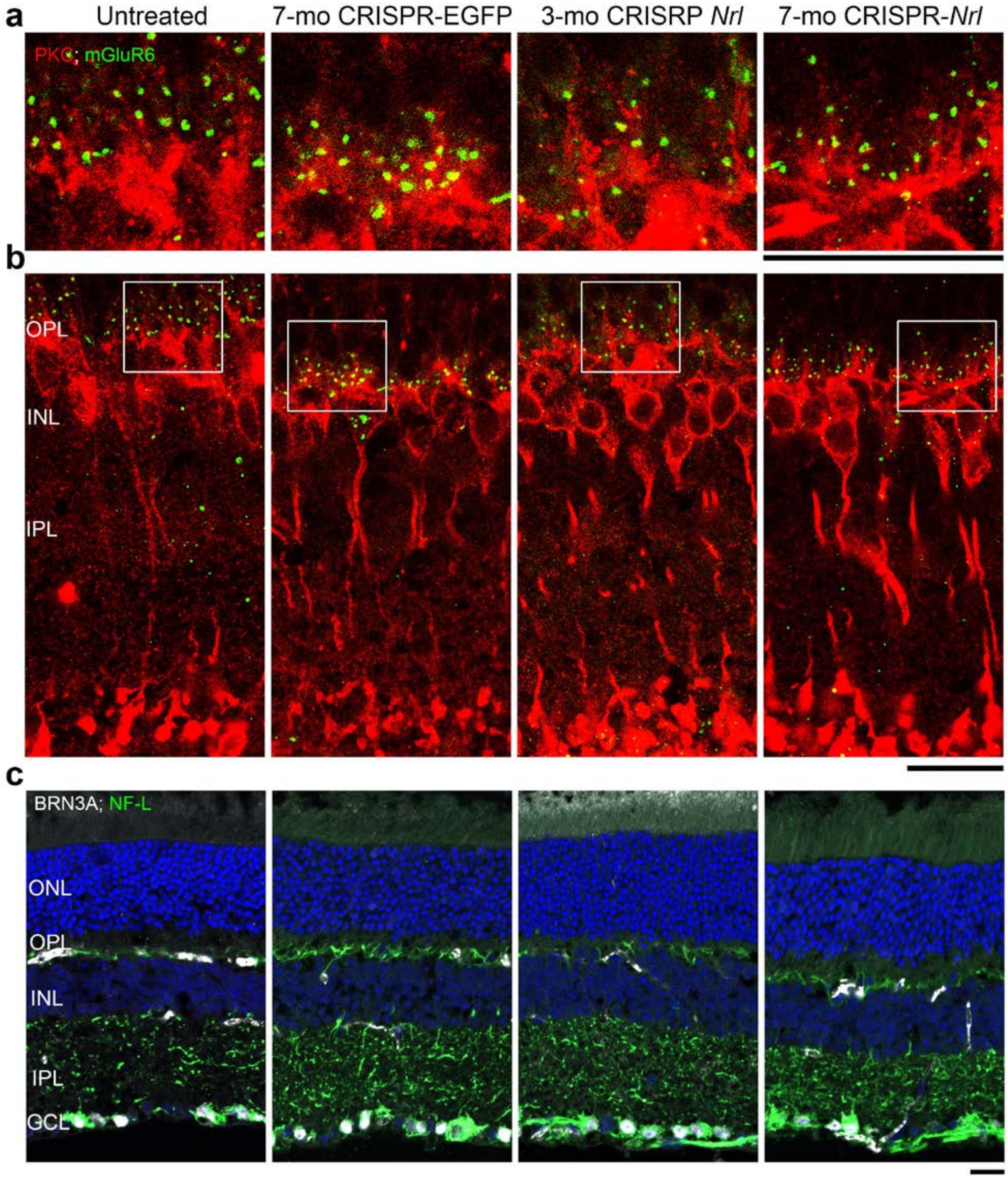

Supplementary Figure 9. Examination of inner retina following CRISPR-*Nrl* treatment. C57/Bl6 mice received subretinal administration of CRISPR-*Nrl* vectors in right eyes and the control CRISPR-EGFP vectors in left eyes at P14. **(a, b)** Immunostaining for mGluR6 (green, arrow) and PKC (red) at 3 months and 7 months of age. **(c)** Immunostaining for BRN3A (white) and Neurofilament Light (NF-L; green). ONL, outer nuclear layer; OPL, outer plexiform layer; INL, inner nuclear layer; IPL, inner plexiform layer; GCL, ganglion cell layer. Scale bars: 20 μm.

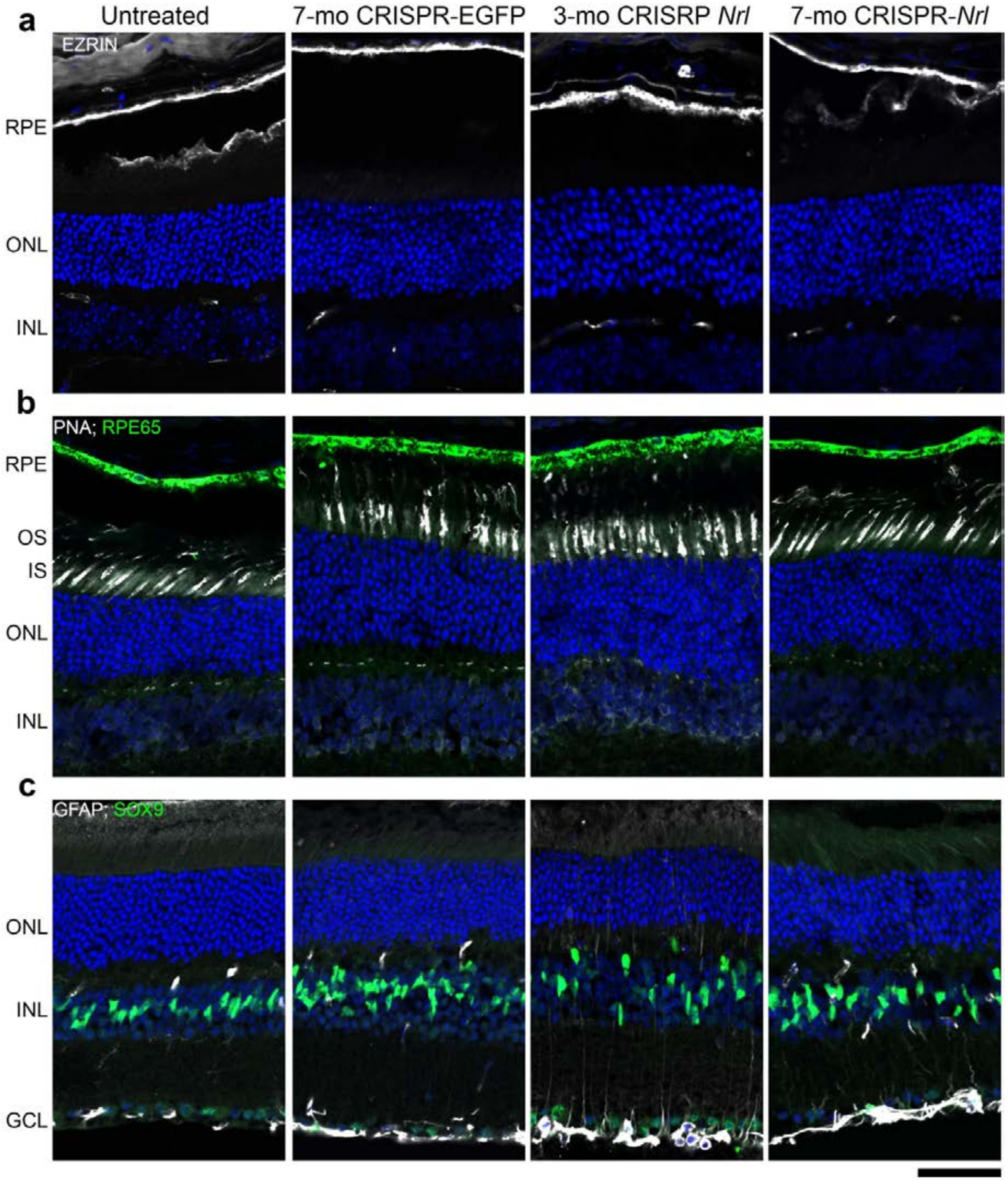

Supplementary Figure 10. Examiniation of RPE and Muller cells following CRISPR-*Nrl* treatment. C57/Bl6 mice received subretinal administration of CRISPR-*Nrl* vectors in right eyes and the control CRISPR-EGFP vectors in left eyes at P14. **(a)** Immunostaining for EZRIN (white) at 3 months and 7 months of age. **(b)** Immunostaining for PNA (white) and RPE65 (green). (**c**) Immunostaining for SOX9 (green) and GFAP (white). RPE, retinal pigment epithelium; OS, outer segments; IS, inner segments; ONL, outer nuclear layer; INL, inner nuclear layer; GCL, ganglion cell layer. Scale bars: 50 μm.

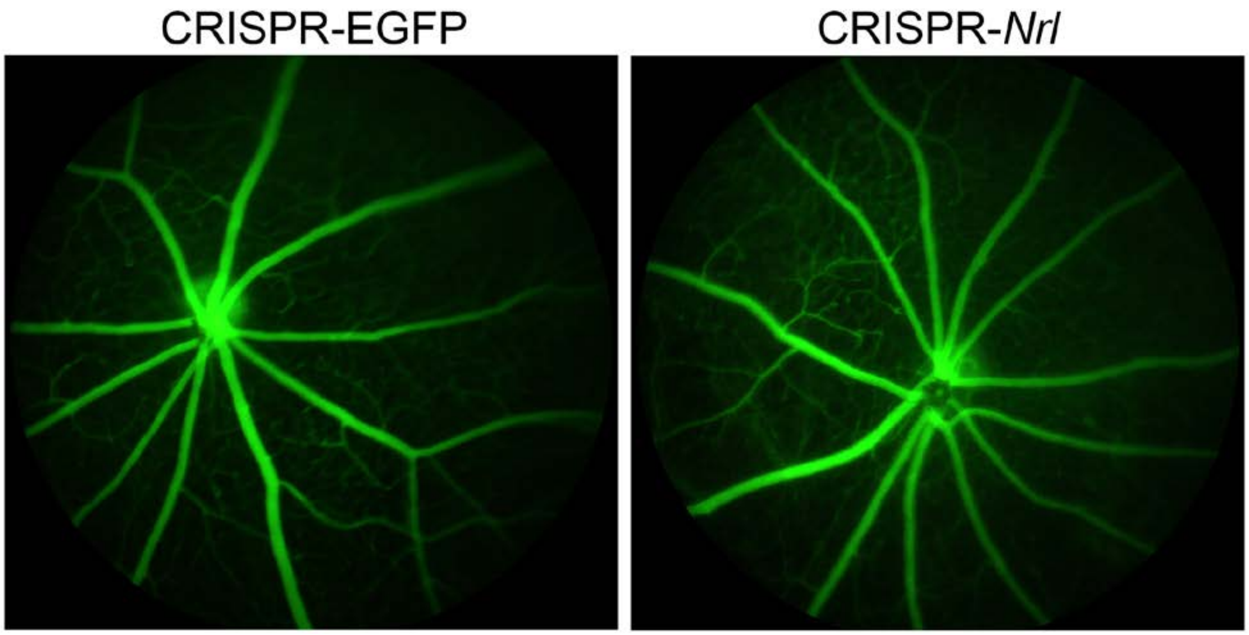

Supplementary Figure 11. Representative fundus fluorescein angiography following CRISPR-*Nrl* treatment. C57bl/6j mice received subretinal administration of CRISPR-*Nrl* (right eyes) or CRISPR-EGFP (control, left eyes) at P14. Fundus fluorescein angiography was conducted on two mice (one male and one female) at 7 months of age. No obvious vascular leakage was observed in CRISPR-*Nrl* treated or control eyes.

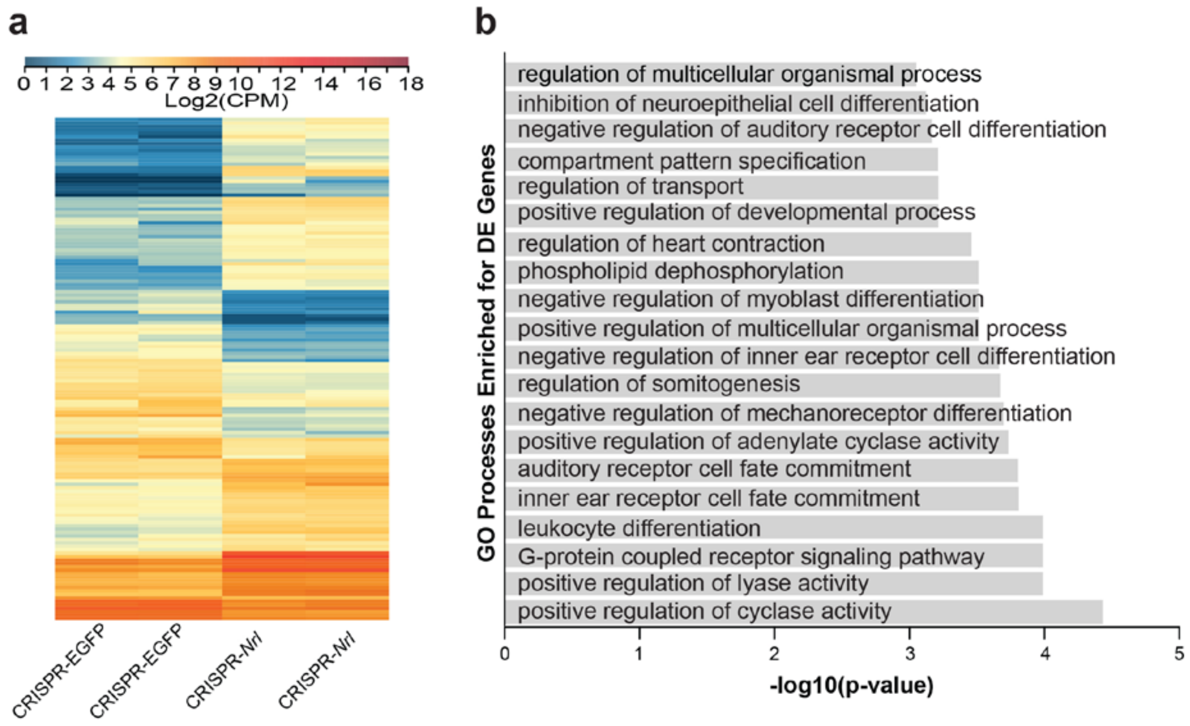

Supplementary Figure 12. Differentially expressed (DE) genes following CRISPR-*Nrl* treatment. C57bl/6j mice received subretinal administration of CRISPR-*Nrl* or CRISPR-EGFP (control) vectors at P14. Two months later, deep RNA sequencing (RNAseq) was performed using total RNA extracted from FACS-enriched tdTomato-expressing retinal cells. Each sample for RNAseq contained RNA from two mouse retinas. (**a**) Expression heatmap of 146 DE genes (fold-change ≥ 2 & adjusted p-value <= 0.05) between control (CRISPR-EGFP treated) cells and CRISPR-*Nrl* treated cells shows distinct expression patterns. Data from two samples per treatment are compared. (**b**) Significant gene ontology (GO) terms enriched for DE genes as identified using GOrilla web tool.

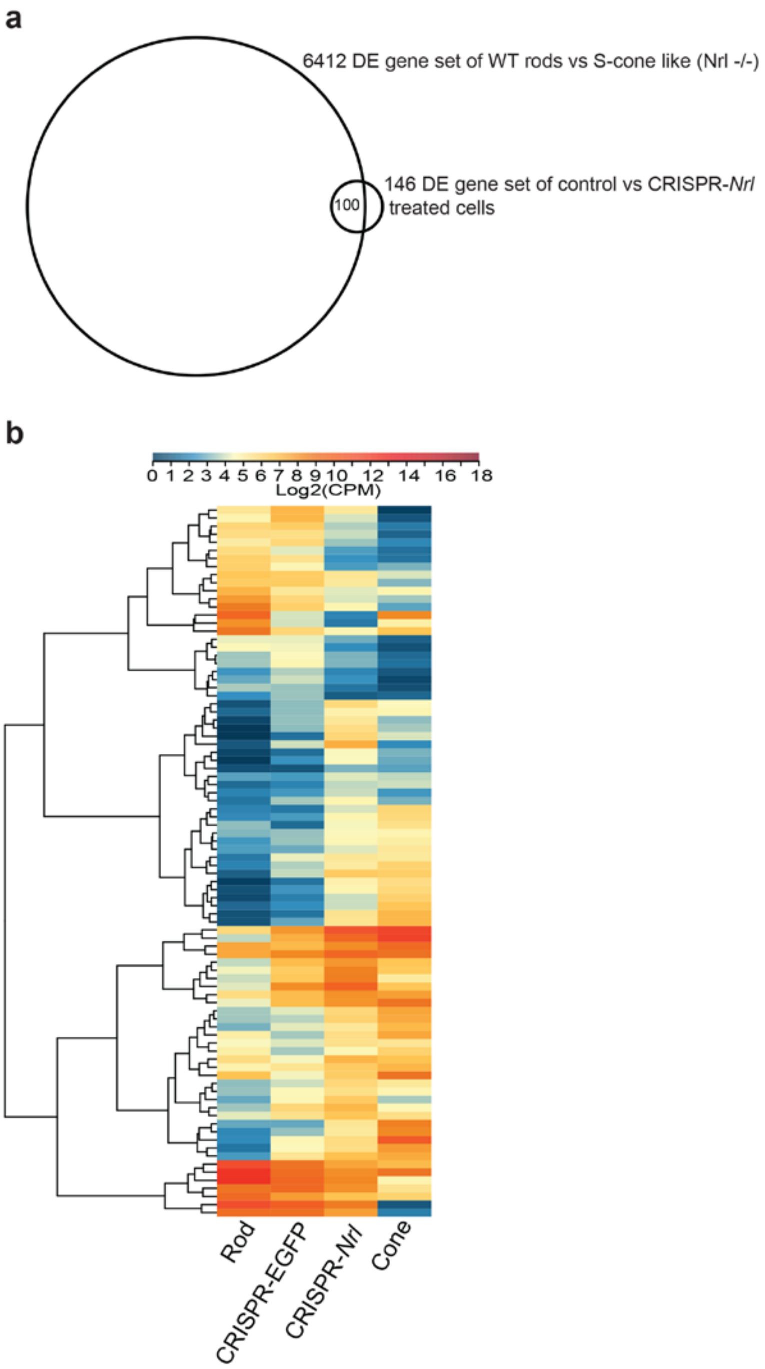

Supplementary Figure 13. Expression alterations in photoreceptors following CRISPR-*Nrl* treatment. (**a**) Venn diagram showing overlap of significant DE genes between mature rods *vs* S-cone like (*Nrl*-/-) cells and CRISPR-EGFP treated *vs* CRISPR-*Nrl* treated postmitotic photoreceptors. One hundred genes were common between the two groups. (**b**) Expression heatmap for 88 of 100 genes that had similar up or down expression trends between the two groups. Biological replicate data was averaged in this analysis.

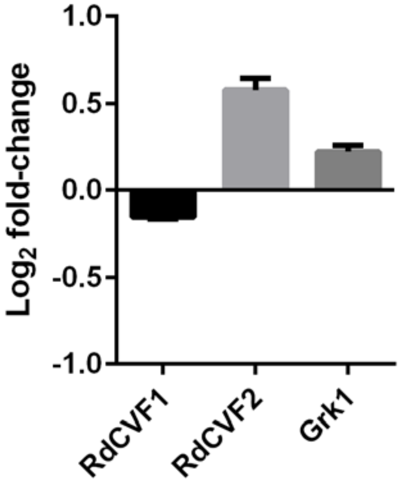

Supplementary Figure 14. Expression alterations of three genes following CRISPR-*Nrl* treatment. C57bl/6j mice received subretinal administration of CRISPR-*Nrl* or CRISPR-EGFP (control) vectors at P14. RNA sequencing analysis of flow-sorted tdTomato-expressing cells was conducted at 2 months post vector injection. Fold differences in expression of rod-derived cone viability factor (*RdCVF*), rod-derived cone viability factor-2 (*RdCVF2*) and rhodopsin kinase (*Grk1*) genes between CRISPR-*Nrl* and CRISPR-EGFP treated eyes are shown.

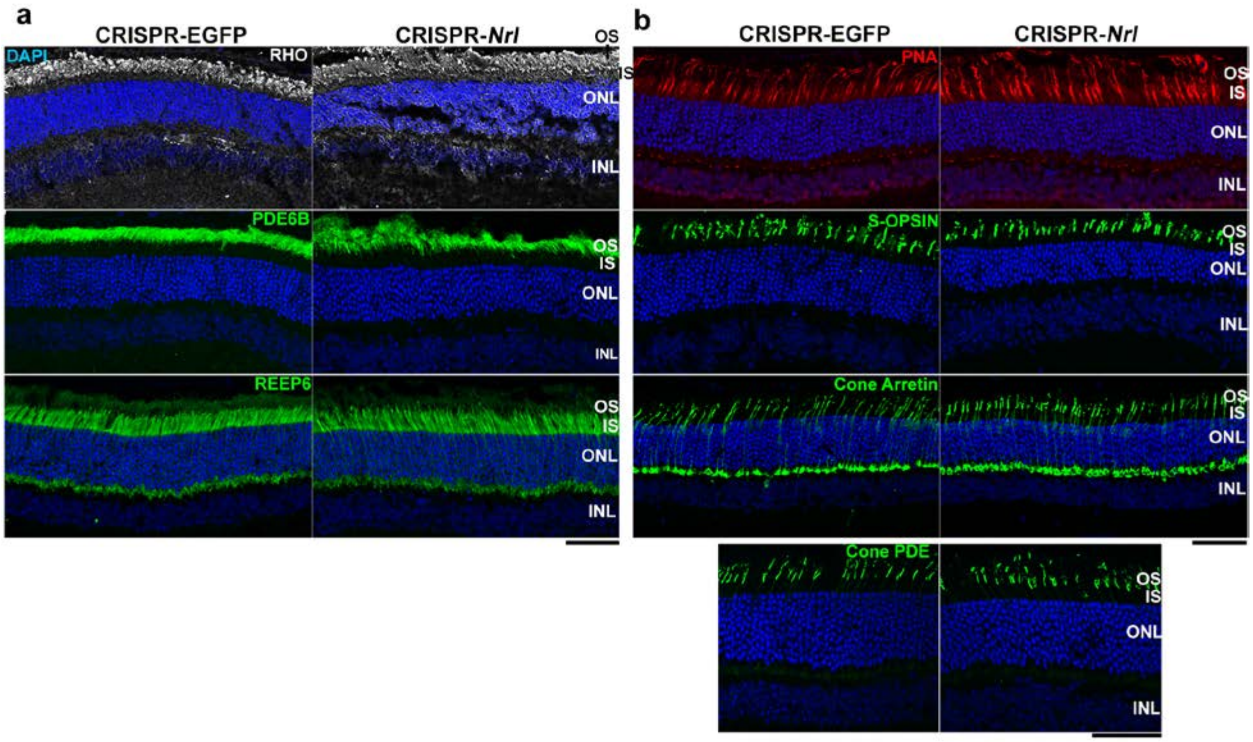

Supplementary Figure 15. Changes of photoreceptor-specific proteins following CRISPR-*Nrl* treatment. C57/Bl6 mice received subretinal administration of CRISPR-*Nrl* vectors in right eyes and the control CRISPR-EGFP vectors in left eyes at P14. Four mice, including both genders, were euthanized at P90 for immunostaining. Representative images are shown. **(a)** Immunostaining for rod-specific proteins including rhodopsin, PDE6β and REEP6 in retinal sections of P90 mice. **(b)** Immunostaining for cone-specific proteins including S-opsin, cone arrestin and cone PDE. PNA staining that labels cone outer segments (OS) and inner segments (IS) was also conducted. Dapi staining is shown in blue. Scale bars, 50 μm.

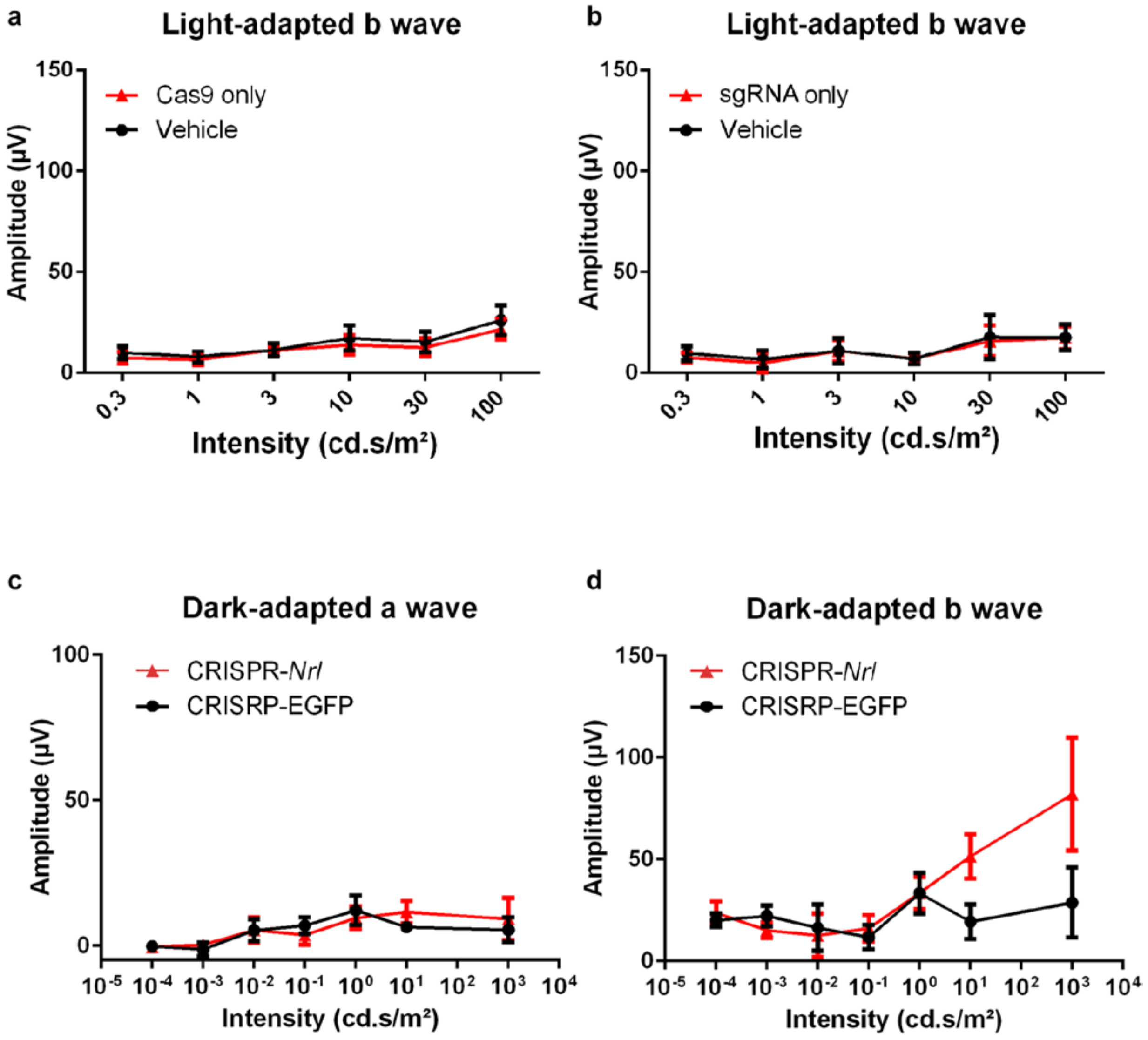

Supplementary Figure 16. ERG of *Rho*$^{-/-}$ mice receiving AAV vector treatment. Mice received subretinal administration of vectors at P14. AAV-Cas9 **(a)** or AAV-sgRNA-*Nrl* **(b)** was injected to the right eyes and vehicle was injected to the left eyes. Light-adapted ERG was conducted at P90. Error bars show SEM and the significance between vector and vehicle treated eyes was calculated using two-tailed paired t-test. No significant differences (P>0.05) were observed for ERG amplitude between the right eyes and left eyes, indicating that the Cas9 vector or the sgRNA vector only could not rescue the cone function in the mice (n=6 for Cas9 vector injected mice; n=4 for sgRNA vector injected mice. Both gender of mice were used). In another cohort (same cohort as shown in Figure 5; n=6, recording of dark-adapted ERG from one mouse of this cohort was not conducted), mice received CRISPR-*Nrl* treatment in right eyes and the control CRISPR-EGFP treatment in left eyes. ERG was conducted at P70. Amplitudes of dark-adapted a wave **(c)** and b wave **(d)** are shown. Error bars show SEM and the significance between CRISPR-*Nrl* and CRISPR-EGFP treated eyes was calculated using two-tailed paired t-test. No significant differences were observed (P>0.05).

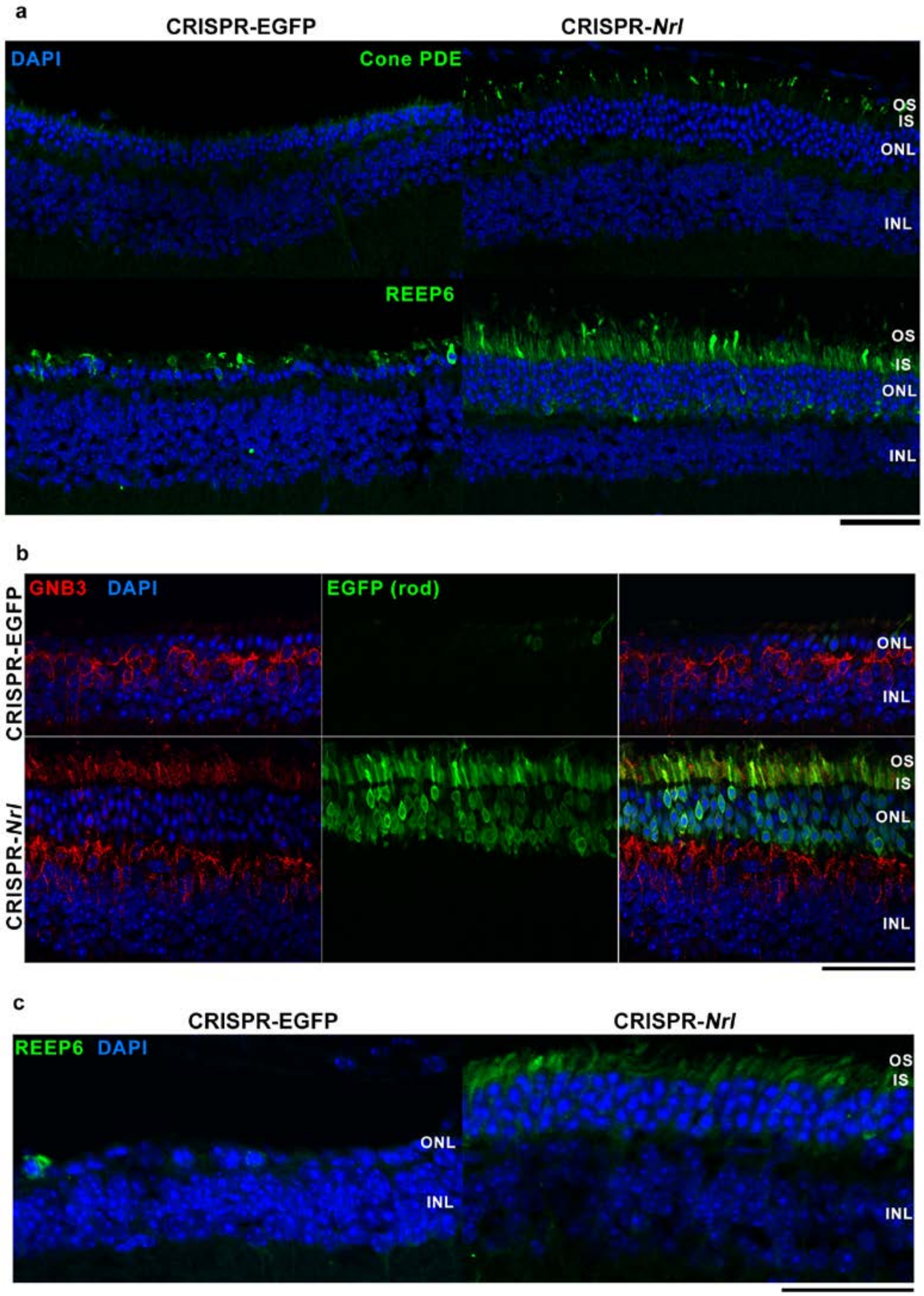

Supplementary Figure 17. Rod or cone-specific markers in diseased mouse models following CRISPR-*Nrl* treatment. Mice received CRISPR-*Nrl* vector treatment in right eyes and the control CRISPR-EGFP vector treatment in left eyes at P14. They were euthanized at P90 and the retinas were collected for immunofluorescence analyses. **(a)** Immunostaining for cone PDE and REEP6 in an *Rho*$^{-/-}$ mouse. **(b)** Immunostaining for GNB3 in an *Rd10/Nrl-L-GFP* mouse. EGFP-expressing cells indicate rod origin. **(c)** Immunostaining for REEP6 in an *RHO P347S* mouse. Scale bar, 50 μm**.**

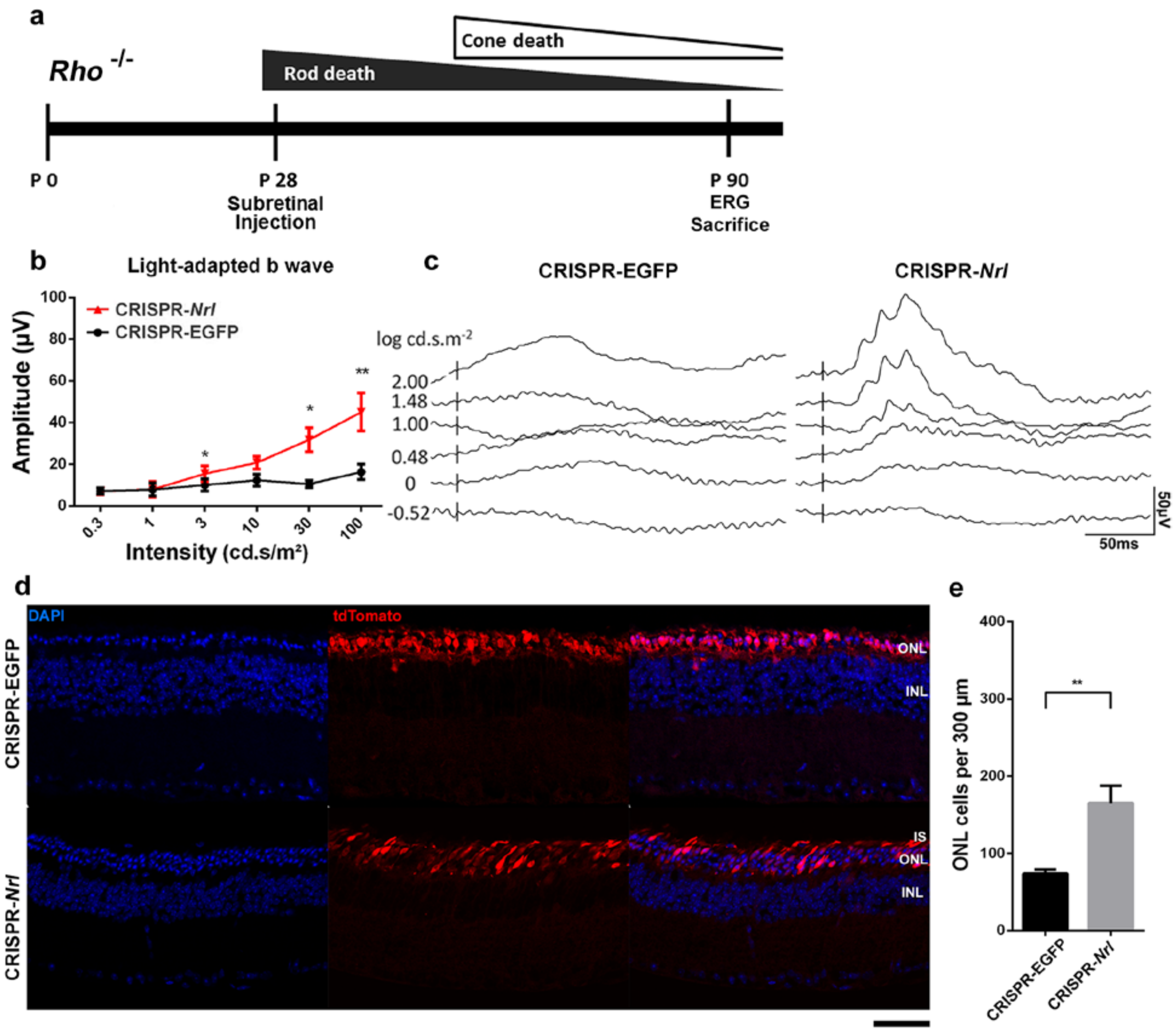

Supplementary Figure 18. Rescue of retinal degeneration following delayed CRISPR-*Nrl* treatment. (**a**) Time course of photoreceptor degeneration in *Rho*$^{-/-}$ mouse (upper) and timeline for CRISPR/Cas9 mediated *Nrl* knockdown experiments (lower). Mice including both genders received CRISPR-*Nrl* vector treatment in right eyes and the control CRISPR-EGFP vector treatment in left eyes at P28. (**b**) ERG analysis at P90 demonstrated significantly larger amplitudes of light-adapted b-wave in response to increasing intensities of flash stimuli in CRISPR-*Nrl* treated eyes than control eyes (n=6, including both genders). (**c**) Representative ERG waveforms from a single mouse at P90. (**d**) Fluorescent microscopy of retina sections reveals thicker outer nuclear layer (ONL) in CRISPR-*Nrl* treated eyes than control eyes. Tdtomato (red) indicates photoreceptors transduced with AAV-sgRNA vectors. Scale bar, 50 µm**.** **(e)** Quantification of ONL cells in 300-µm segments of retina (n=3). Error bars show SEM and the significance between the CRISPR-*Nrl* and the CRISPR-EGFP treated eyes was calculated using two-tailed paired t-test. * $p<0.05$; ** $p<0.01$.

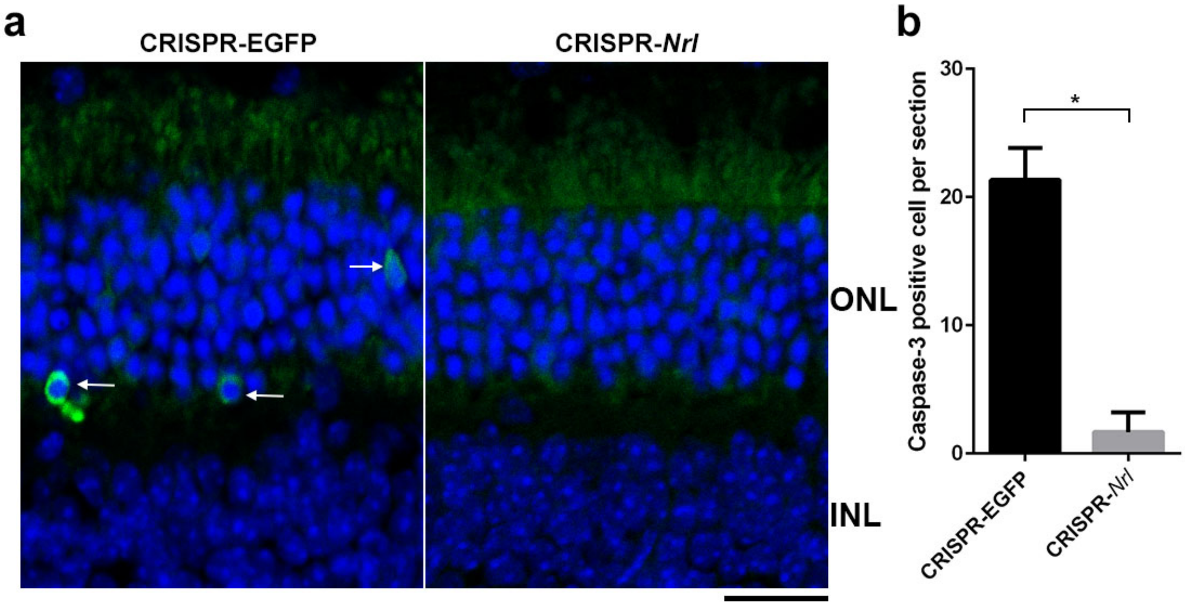

Supplementary Figure 19. Inhibition of caspase-3 mediated apoptosis by CRISPR-*Nrl*. *RHO P347S* mice including both genders received subretinal administration of CRISPR-*Nrl* vectors in right eyes and the control CRISPR-EGFP vectors in left eyes at P14. **(a)** Immunostaining for cleaved caspase-3 (green) at 1 month post treatment. Scale bars: 20 μm. (**b**) Quantification of caspase-3 positive cells in retina sections (n = 3). Error bars show SEM and the significance between the CRISPR-EGFP and CRISPR-*Nrl* treated eyes was calculated using two-tailed paired t-test. * $p<0.05$.

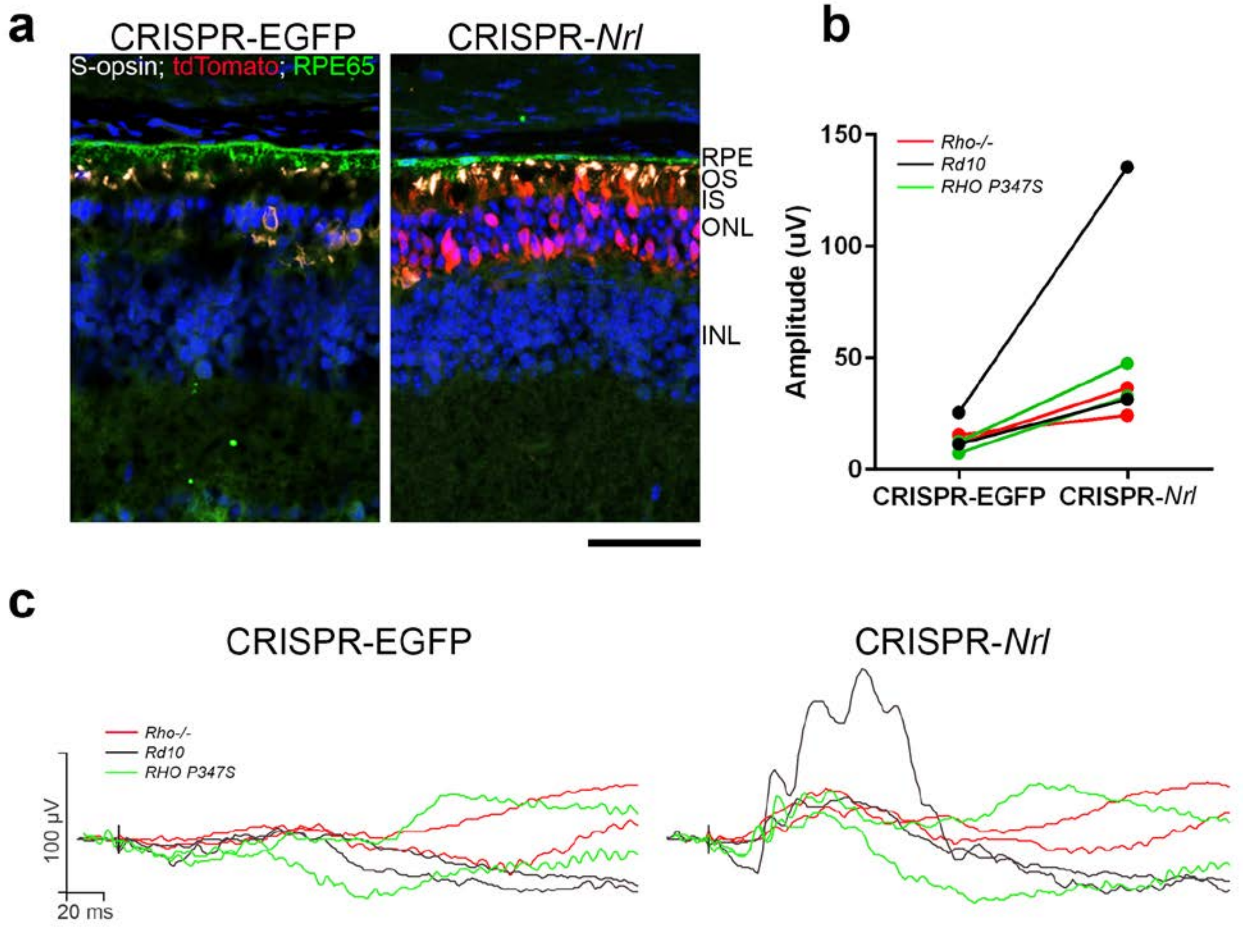

Supplementary Figure 20. Rescue of retinal degeneration at 4 months of age. Mice including both genders received CRISPR-*Nrl* vector treatment in right eyes and the control CRISPR-EGFP vector treatment in left eyes at P14. (**a**) Representative immunofluorescence images of a *Rho*$^{-/-}$ mouse revealing normal RPE integrity, better preserved ONL and S-opsin expression in CRISPR-*Nrl* treated eye than the control eye. Scale bar: 50 µm. (**b** and **c**) Comparison of photopic ERG amplitude (**b**) and wave form (**c**) between CRISPR-*Nrl* treated eyes and CRISPR-EGFP treated eyes in *Rho*$^{-/-}$ (red line, n=2), *Rd10* (black line, n=2) and *RHO P347S* mice (green line, n=2) with the flash stimulus of 100 cd.s.m^{-2}.

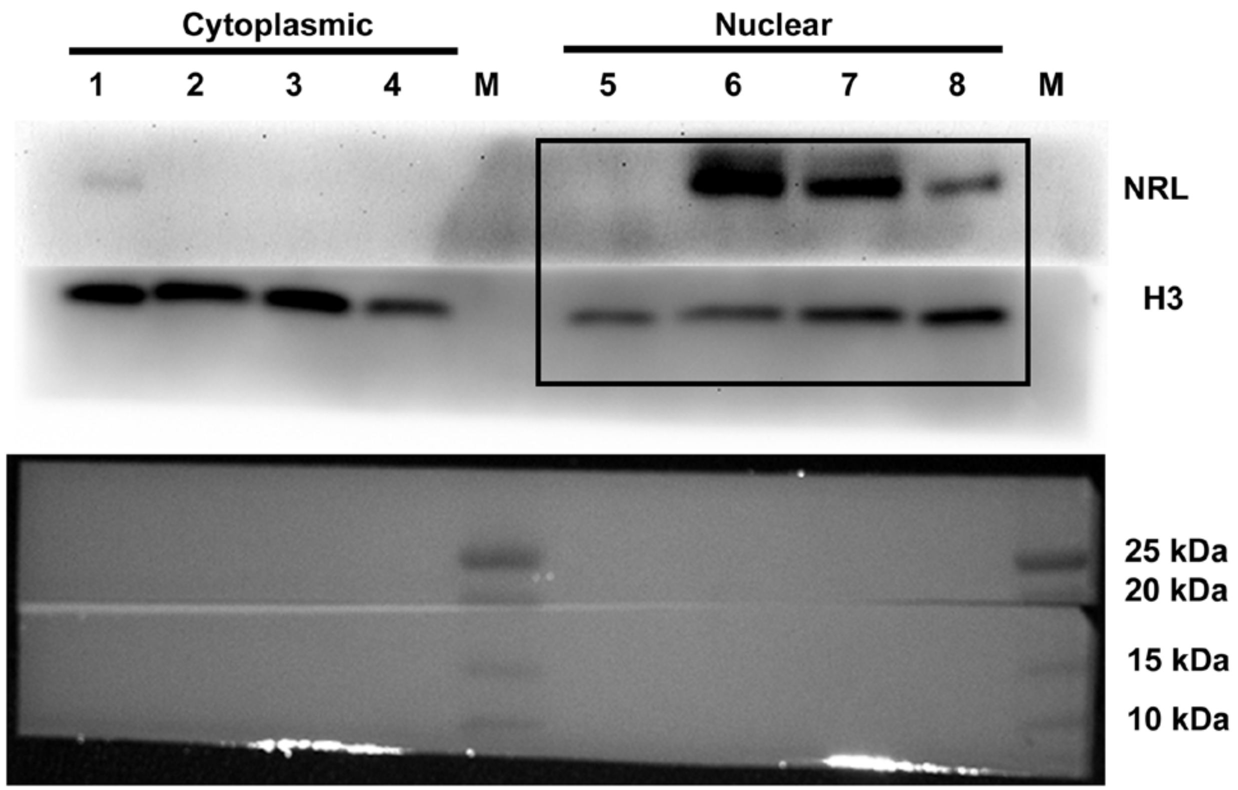

Supplementary Figure 21. Uncropped scans of the Western blot images shown in Figure 3f. Protein extracted from cytoplasm (lane 1, 2, 3 and 4) and nucleus (lane 5, 6, 7 and 8) of retinas from *Nrl*-KO (1 and 5), C57bl/6j (2 and 6), CRISPR-EGFP vector treated (3 and 7) and CRISPR-*Nrl* vector treated C57bl/6j mice (4 and 8) were analyzed. H3: nuclear loading control Histone H3.

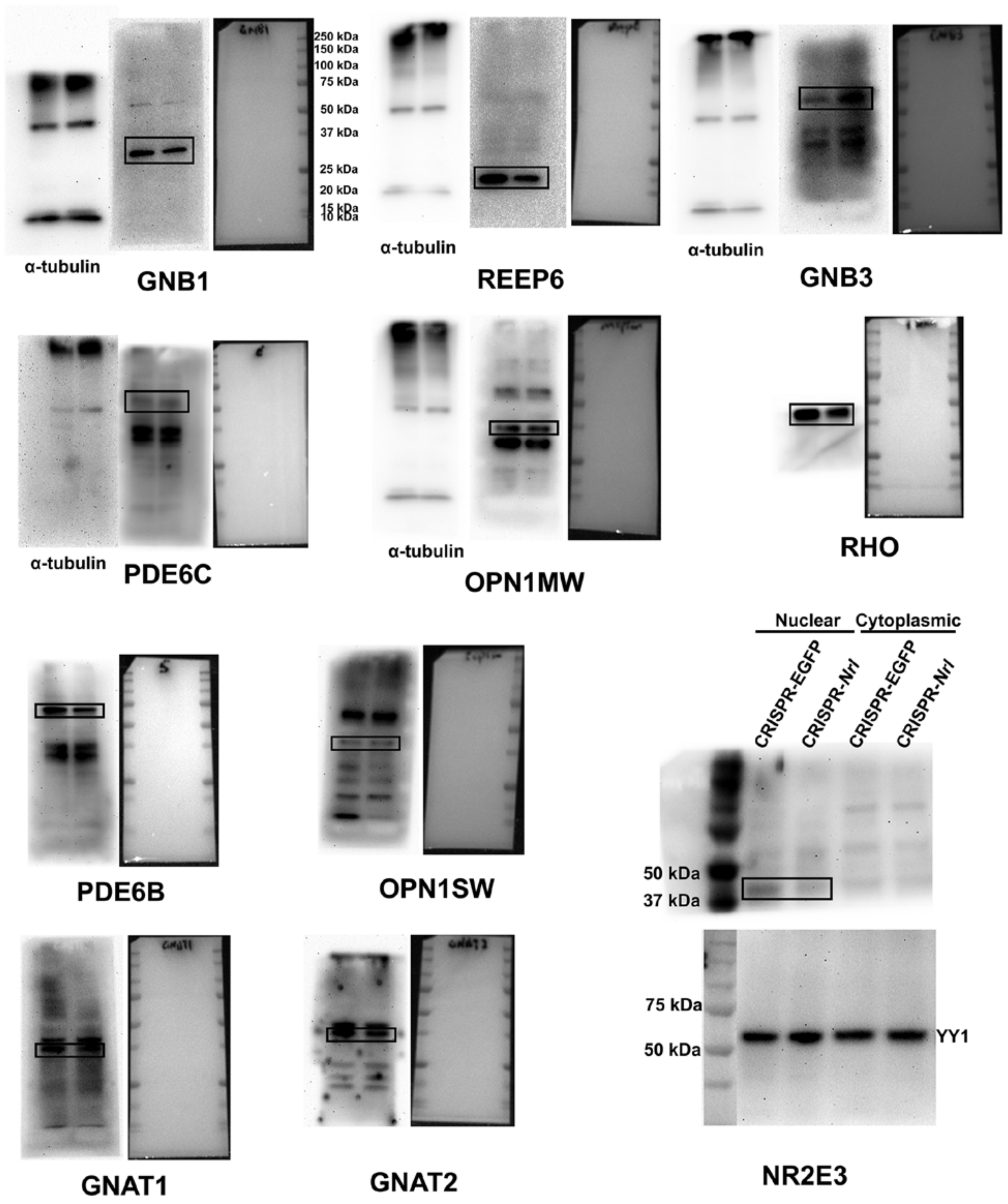

Supplementary Figure 22. Uncropped scans of the Western blot images shown in Figure 5d.

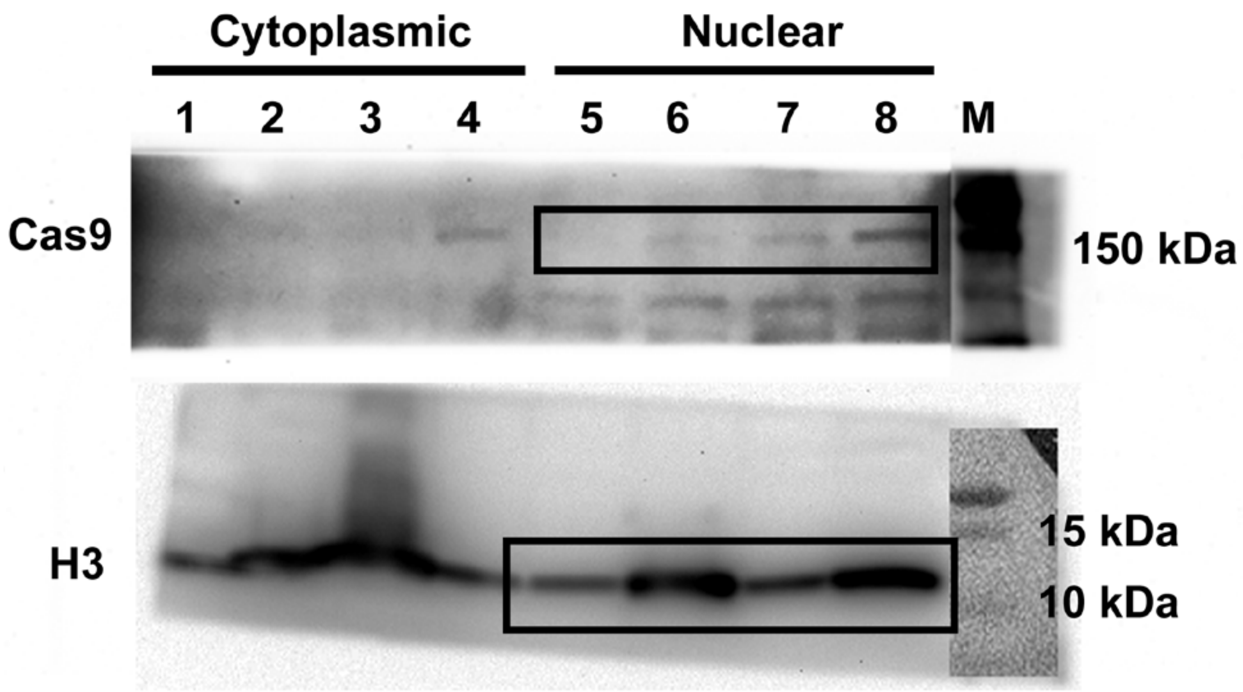

Supplementary Figure 23. Uncropped scans of the Western blot images shown in Supplementary Figure 1b. Protein extracted from cytoplasm (lane 1, 2, 3 and 4) and nucleus (lane 5, 6, 7 and 8) of retinas from wildtype C57bl/6j mice receiving AAV-Cas9 vectors at doses of 0 (1 and 5), $5x10^8$ (2 and 6), $1x10^9$ (3 and 7) and $5x10^9$ (4 and 8) vg per eye were analyzed. H3: nuclear loading control Histone H3.

Supplementary Table 1. sgRNA target candidates and PCR primers for SURVEYOR assays

ID	Sequence (5' to 3')	PAM	Ref.	Primer Name	Primer Sequence (5' to 3')	Size of PCR Product (bp)	Estimated fragment size (bp) after SURVEYOR assay
**NrlTarget1**	GAGCCTTCTGAGGGCCGATC	TGG		Nrl_test_F<br>Nrl_test_R	GGATCCATGGCTTTCCCTCCCAG<br>CACACACCTCTTCCTCTGACTCGAG	726	98 & 628
**NrlTarget2**	**GTATGGTGTGGAGCCCAACG**	**AGG**					121 & 605
**NrlTarget3**	AGGCACCGAGCTGTATGGTG	TGG					133 & 593
**NrlTarget4**	GGTGGCGAGGCCCCTAGGCC	AGG					203 & 523
**NrlTarget5**	CCTGGAGGAGCTATATTGGC	TGG					226 & 500
**EGFPTarget1**	GAGCTGGACGGCGACGTAAA	CGG		EGFP_test_1F<br>EGFP_test_1R	ATGGTGAGCAAGGGCGAG<br>GGCGGACTTGAAGAAGTCGT	264	68 & 196
**EGFPTarget2**	CAACTACAAGACCCGCGCCG	AGG		EGFP_test_2F<br>EGFP_test_2R	CGACGTAAACGGCCACAAG<br>ACCATGTGATCGCGCTTCTC	597	269 & 328
**EGFPTarget3**	**CGCGCCGAGGTGAAGTTCGA**	**GGG**		EGFP_test_2F<br>EGFP_test_2R	CGACGTAAACGGCCACAAG<br>ACCATGTGATCGCGCTTCTC	597	282 & 315
**EGFPTarget4**	AGCACGGGGCCGTCGCCGAT	GGG		EGFP_test_3F<br>EGFP_test_3R	GTGAAGTTCGAGGGCGACA<br>CTTGTACAGCTCGTCCATGCC	381	150 & 231
**EGFPTarget5**	CCATCGGCGACGGCCCCGTG	GGG		EGFP_test_3F<br>EGFP_test_3R	GTGAAGTTCGAGGGCGACA<br>CTTGTACAGCTCGTCCATGCC	381	152 & 229
**EGFPTarget6**	GGTGGTGCAGATGAACTTCA	GGG	1	EGFP_test_1F<br>EGFP_test_1R	ATGGTGAGCAAGGGCGAG<br>GGCGGACTTGAAGAAGTCGT	264	136 & 128
**EGFPTarget7**	GGCCACAAGTTCAGCGTGTC	CGG	2	EGFP_test_1F<br>EGFP_test_1R	ATGGTGAGCAAGGGCGAG<br>GGCGGACTTGAAGAAGTCGT	264	89 & 175
**EGFPTarget8**	GAAGTTCGAGGGCGACACCC	TGG	2	EGFP_test_2F<br>EGFP_test_2R	CGACGTAAACGGCCACAAG<br>ACCATGTGATCGCGCTTCTC	597	293 & 304

Supplementary Table 2. On- and Off-target analysis

Name	Sequence	PAM	Gene	Chr	#MM*	NHEJ% of ~3 month			NHEJ% of ~10 month		
						Treated	Control	Untreated	Treated	Control	Untreated
On-target	GTATGGTGTGGAGCCCAACG	AGG	Nrl (ENSMUSG00000040632)	chr14:+55522354	0	98.39969	0.714286	0.395257	93.20267	0.109324	0.1835
OT1	GTATGGTGTGGtGCCCAACt	GAG	None	chr9:+20579047	2	0.526816	0.483823	0.551229	0.252908	0.404594	0.246002
OT2	GcAgGGTGaaGAGCCCAACG	CAG	None	chrX:+140278339	4	0.139437	0.203892	0.12422	0.364647	0.418692	0.341634
OT3	tcATtGTGTGGAGCCCAAcG	GGG	None	chr8:-115610670	4	0.113817	0.199117	0.171572	0.967142	1.388456	0.98219
OT4	GctTtGTGTGGAtCCCAACG	TGG	None	chr15:-64161229	4	NA	NA	0.86133	0.054451	0.149477	0.092428
OT5	GTgTGGcGTGaAGgCCAACG	AGG	Tjp3(ENSMUSG00000034917)	chr4:-137753970	4	0.619595	0.60444	0.634414	0.458535	0.459184	0.339919
OT6	GTAcGGcGTGGAGaCCtACG	GGG	Alpl (ENSMUSG00000028766)	chr1:-17097238	4	0.178439	0.592417	0.179099	0.384388	0.394388	0.682701
OT7	GcATGGTGTGcAGCCCAgaG	CAG	Jph1 (ENSMUSG00000042686)	chr17:+23737599	4	0.178439	0.216884	0.114556	4.603899	6.944444	4.784537
OT8	GgATGcTGTGGAGtCCAAgG	TGG	Shank1 (ENSMUSG00000038738)	chr7:-44353726	4	NA	NA	NA	1.608579	2.175606	1.688769
OT9	GTgTGGTcTGGAGtCCAgCG	TGG	Fam173b (ENSMUSG00000039065)	chr15:-31616952	4	0.132436	0.211762	0.196861	NA	0.4769	0.373715
OT10	GTgTGGTGTGGAGCCCAgaa	CAG	Ppp1r1a (ENSMUSG00000022490)	chr15:-103531410	4	0.107641	0.106828	0.113244	0.080915	0.124208	0.093505

* #MM=Number of Mismatches

Supplementary Table 3. Primers for SURVEYOR assay and/or sequencing

Purpose	Name	Sequence (5' to 3')	Product Size (bp)	Estimated fragment size (bp) after SURVEYOR assay
**In vivo EGFP knockdown**	Ontarg_EGFP_T3_F	TCGTGACCACCCTGACCTAC	337	163 & 174
	Ontarg_EGFP_T3_R	TCGATGTTGTGGCGGATCTT		
**In vivo *Nrl* knockdown**	Ontarg_Nrl_T2_F	GCAGACAGCCTCTCAGTGTT	343	157 & 186
	Ontarg_Nrl_T2_R	CTCAGCCCGAGAACCTCATC		
	Offtarg_NrlT2_OT1F	CAGATTGGAGGGTGAGGCAA	471	-
	Offtarg_NrlT2_OT1R	GCTCCAAGGTTCATTCTGCAC		
	Offtarg_NrlT2_OT2F	GGAGACAGGGGCAGGGTT	295	-
	Offtarg_NrlT2_OT2R	GGCTATGTGTGCGTGAGAGT		
	Offtarg_NrlT2_OT3F	GCCCTTTCAGCTTTTCTGCC	413	-
	Offtarg_NrlT2_OT3R	TACCTCCCAGCCACTTCTCA		
	Offtarg_NrlT2_OT4F	GAAAGCAGGGCCTAGGTGTT	310	-
	Offtarg_NrlT2_OT4R	AGGAGCTGGAGTGTGGTACA		
	Offtarg_NrlT2_OT5F	CACCTACCTCTTGCGGTCAT	467	-
	Offtarg_NrlT2_OT5R	TTCCCGGCCCCAGGAATTAT		
	Offtarg_NrlT2_OT6F	AACCTTGAGTTCTGCCAGGG	352	-
	Offtarg_NrlT2_OT6R	GATGGCTCACCAGCATCCTT		
	Offtarg_NrlT2_OT7F	AGGTTCGACTTCGACGATGG	485	-
	Offtarg_NrlT2_OT7R	ACTCCCCAGATGTTTTGGCG		
	Offtarg_NrlT2_OT8F	TTCGAAAAGCCAGAGTCGCC	355	-
	Offtarg_NrlT2_OT8R	GCAACGTAGGTGTCCAGGAG		
	Offtarg_NrlT2_OT9F	GTGTTGAGGGTCCTAACGGC	323	-
	Offtarg_NrlT2_OT9R	CGGAAGATTAGCAGCGGTGA		
	Offtarg_NrlT2_OT10F	CGGCAAGGTAGATCTGCTCC	384	-
	Offtarg_NrlT2_OT10R	TCCTCTCCGCAGTCACCTAA		

Supplementary Table 4. Primary antibodies

Antibody	Aplication	Description	Dilution	Source
**NRL**	WB	Rabbit polyclonal	1/2000	Custom made and kept in lab
	IF	Rat polyclonal	1/500	Custom made and kept in lab
**CRX**	IF	Rabbit polyclonal	1/500	Santa Cruz; sc-30150
**CAS9**	WB	Mouse monoclonal	1/1000	Diagenode Inc; 4G10
**Histone H3**	WB	Rabbit polyclonal	1/2000	Abcam; ab1791
**RHO**	WB,IF	Mouse monoclonal	1/2000 for WB; 1/1000 for IF	Custom made and kept in lab; [3]
**GNAT1**	WB	Rabbit polyclonal	1/2000	Santa Cruz; sc-389
**GNB1**	WB	Rabbit polyclonal	1/2000	Santa Cruz; sc-379
**PDE6B**	WB,IF	Rabbit polyclonal	1/2000 for WB; 1/1000 for IF	Custom made and kept in lab; [4]
**NR2E3**	WB	Rabbit polyclonal	1/2000	Santa Cruz; sc-292264
**YY1**	WB	Rabbit monoclonal	1/2000	Cell Signaling Technology; 2185
**REEP6**	WB,IF	Rabbit polyclonal	1/2000 for WB; 1/1000 for IF	Custom made and kept in lab; [5]
**M-OPSIN**	WB,IF	Rabbit polyclonal	1/2000 for WB; 1/500 for IF	Millipore; AB5404
**S-OPSIN**	WB,IF	Chicken Polyclonal	1/2000 for WB; 1/500 for IF	Custom made and kept in lab; [4]
**GNAT2**	WB	Rabbit polyclonal	1/2000	Santa Cruz; sc-390
**GNB3**	WB,IF	Rabbit polyclonal	1/1000 for WB; 1/500 for IF	Custom made and kept in lab
**PDE6C**	WB,IF	Rabbit polyclonal	1/1000 for WB; 1/500 for IF	Custom made and kept in lab; [4]
**α-tubulin**	WB	Mouse monoclonal	1/3000	DSHB; AA4.3-c; [6]
**PNA**	IF	Alexa Fluor® 647 Conjugated	1/1000	Invitrogen; L32460
**RPE65**	IF	Rabbit polyclonal	1/400	Kindly provided by Dr. T.M. Redmond, National Eye Institute, Bethesda, MD; [7]
**SOX9**	IF	Rabbit polyclonal	1/500	Millipore; AB5535
**BRN3A**	IF	Mouse monoclonal	1/500	Millipore; MAB1585
**mGluR6**	IF	Sheep polyclonal	1/400	Kindly provided by Dr. Wei Li, National Eye Institute, Bethesda, MD
**PKC**	IF	Mouse monoclonal	1/500	Pierce; MA1-46107
**NF-L**	IF	Rabbit polyclonal	1/500	Chemicon; AB9568
**EZRIN**	IF	Mouse monoclonal	1/500	Thermo Scientific; MS-661-P1ABX
**GFAP**	IF	Mouse monoclonal	1/500	Sigma Aldrich; G3893
**Caspase-3, Cleaved**	IF	Rabbit polyclonal	1/400	Cell Signaling Technology; 9661S

Supplementary References

1. Fu Y, Sander JD, Reyon D, Cascio VM, Joung JK. Improving CRISPR-Cas nuclease specificity using truncated guide RNAs. *Nature biotechnology*, (2014).

2. Shalem O*, et al.* Genome-scale CRISPR-Cas9 knockout screening in human cells. *Science* **343**, 84-87 (2014).

3. Li T, Snyder WK, Olsson JE, Dryja TP. Transgenic mice carrying the dominant rhodopsin mutation P347S: evidence for defective vectorial transport of rhodopsin to the outer segments. *Proceedings of the National Academy of Sciences of the United States of America* **93**, 14176-14181 (1996).

4. Sun X*, et al.* Gene therapy with a promoter targeting both rods and cones rescues retinal degeneration caused by AIPL1 mutations. *Gene therapy* **17**, 117-131 (2010).

5. Keeley PW*, et al.* Development and plasticity of outer retinal circuitry following genetic removal of horizontal cells. *The Journal of neuroscience : the official journal of the Society for Neuroscience* **33**, 17847-17862 (2013).

6. Walsh C. Synthesis and assembly of the cytoskeleton of Naegleria gruberi flagellates. *The Journal of cell biology* **98**, 449-456 (1984).

7. Roger JE*, et al.* Preservation of cone photoreceptors after a rapid yet transient degeneration and remodeling in cone-only Nrl-/- mouse retina. *The Journal of neuroscience : the official journal of the Society for Neuroscience* **32**, 528-541 (2012).
